# Regional or general anesthesia for fast-track hip and knee replacement - what is the evidence?

**DOI:** 10.12688/f1000research.7100.1

**Published:** 2015-12-15

**Authors:** Henrik Kehlet, Eske Kvanner Aasvang

**Affiliations:** 1 Section for Surgical Pathophysiology, Rigshospitalet, Copenhagen University Hospital, Copenhagen, Denmark; 2 The Lundbeck Foundation Centre for Fast-Track Hip and Knee Replacement, Copenhagen, Denmark; 3 Anesthesiological Department of the Abdominal Centre and Section for Surgical Pathophysiology, Rigshospitalet, Copenhagen University, Blegdamsvej 9, DK–2100, Denmark

**Keywords:** Regional anesthesia, general anesthesia, hip replacement, knee replacement

## Abstract

Regional anesthesia for knee and hip arthroplasty may have favorable outcome effects compared with general anesthesia by effectively blocking afferent input, providing initial postoperative analgesia, reducing endocrine metabolic responses, and providing sympathetic blockade with reduced bleeding and less risk of thromboembolic complications but with undesirable effects on lower limb motor and urinary bladder function. Old randomized studies supported the use of regional anesthesia with fewer postoperative pulmonary and thromboembolic complications, and this has been supported by recent large non-randomized epidemiological database cohort studies. In contrast, the data from newer randomized trials are conflicting, and recent studies using modern general anesthetic techniques may potentially support the use of general versus spinal anesthesia. In summary, the lack of properly designed large randomized controlled trials comparing modern general anesthesia and spinal anesthesia for knee and hip arthroplasty prevents final recommendations and calls for prospective detailed studies in this clinically important field.

## Introduction and context

The discussion on the optimal anesthetic technique for most surgical procedures regarding the use of regional anesthetic versus general anesthetic techniques has been going on for decades. In hip and knee replacement, several randomized trials performed several decades ago were in favor of spinal or epidural analgesia ^1, 2^. This is probably explained by the positive physiological effects of the provided afferent blockade with better initial pain relief, a reduced endocrine metabolic response, and sympathetic blockade with less blood loss and increased leg blood flow, all resulting in reduced cardiopulmonary and thromboembolic morbidity, but at the potential cost of reduced capability for early postoperative mobilization, urinary bladder dysfunction, and rare but potentially severe neurological complications.

In recent years, several large epidemiological studies based on the large US databases (Premier and National Surgical Quality Improvement Program) have supported the old studies by demonstrating less postoperative morbidity when using regional anesthetic techniques ^2– 7^. However, these large-scale studies have little or no information on the type of general anesthesia, perioperative pain management, or details on the provided regional anesthetic technique. Furthermore, information on the care principles regarding the use of the fast-track methodology ^8^ has not been provided, and most importantly comparisons have not been made on a randomized basis, thereby introducing a potential large selection bias. More recent reviews from randomized studies, but again without exact data on care principles and pain management, have questioned the benefits of regional anesthesia versus general anesthesia ^9^ or even a higher risk for cardiovascular complications with neuraxial anesthesia ^10^. In conclusion, the jury is still out for conclusive evidence for the optimal choice of regional versus general anesthetic techniques for knee and hip arthroplasty.

The goal of this brief review is to update the literature and discuss the potential for a more balanced view regarding the choice of anesthesia for hip and knee arthroplasty within a fast-track setup ^8^, in which length of stay (LOS) before going home is now usually between 1 and 3 days ^11– 13^ and in which previous data have not shown firm differences between the two anesthetic techniques or in selected patient groups ^2– 7, 9^.

## Recent advances and topics of interest

Recently, two relatively small randomized studies (n = 120 in each) have compared modern target-controlled infusion with propofol and remifentanil versus a conventional spinal anesthesia (without opioids), within a fast-track setup and expected LOS of around 2 days, and with additional multimodal oral opioid-sparing analgesia ^14, 15^. These two studies showed no clinically relevant differences in functional recovery outcomes, LOS, or side effects regarding urinary bladder dysfunction and mobilization. However, after the initial few postoperative hours with residual effects of the spinal anesthesia, there were minor but probably not clinically relevant advantages in analgesia and opioid requirements in the general anesthesia group. Though of interest because of the modern general anesthesia technique and fast-track setup, these studies obviously cannot answer the important question about safety issues and potential differences in postoperative morbidity between the two anesthetic techniques but merely serve as a stimulus to perform the required large comparative studies.

## Type of regional anesthesia

Epidural analgesia should not be used routinely in fast-track total hip arthroplasty (THA) or total knee arthroplasty (TKA) because of the limited analgesic effect, especially in comparison with local wound infiltration (local infiltration analgesia, or LIA) in TKA ^16^ combined with the potential for adverse effects such as urinary retention, pruritus, hypotension, and motor blockade ^17, 18^, all of which delay recovery.

Spinal anesthesia should be performed using only local anesthetics, as intrathecal opioids increase the risk of urinary retention, pruritus, and respiratory depression ^19^ unless low doses (less than 200 µg) are used, and may not have superior analgesic efficacy compared with LIA in TKA ^16, 20^. Recommendations on the optimal dosage of the various types of local anesthetics are beyond the scope of this review. However, one of the challenges in spinal anesthesia for fast-track THA and TKA is optimal titration to provide sufficient analgesia during surgery without a recovery delay due to adverse effects, including impairment to motor function. This requires a strict focus on time spent for preparation and surgery, where a dosage of local anesthetic that is too low may result in the need for supplemental intravenous analgesics (opioids) or conversion to general anesthesia during surgery. However, doses as low as 5 mg bupivacaine have been proven sufficient for 60-minute procedures without the need for conversion to general anesthesia, but in combination with femoral and sciatic nerve blocks ^21^ and their possible negative implications for motor function and recovery.

## Complications to the anesthesia *per se*

General anesthesia imposes various degrees of potential risks related mainly to airway management and respiration (dental and oral soft tissue injuries, vocal cord trauma, barotrauma from positive pressure ventilation, aspiration, and so on) and circulation (negative inotropic and chronotropic cardiac effects from anesthetics) ^4, 6, 7^. Complications to spinal and epidural anesthesia also include hypotension due to the vasodilatory effect of the sympathetic blockade, in addition to the rare but potentially serious risk of compressive neuraxial hematoma. However, this occurs after spinal anesthesia in a maximum of 1 out of 775,000 procedures but in 1 out of 9000 to 1 out of 26,000 epidural procedures, again emphasizing that epidural should not be used ^22– 24^. The occurrence of neurological deficits from neuraxial blockade should be held against the overall risk of nerve injury after THA (0.08% to 1.7% in larger series) and TKA (0.3% to 0.9% in larger series) ^25^. Comparison of the risk profiles for adverse events after general and spinal anesthesia needs to take into account that the choice of anesthesia and subsequent complications are affected mainly by patient characteristics. This is a main bias in the current large nationwide database studies reporting significantly higher complication rates after general anesthesia ^4, 6, 7^. There is general agreement that neuraxial anesthesia may lead to bladder dysfunction in the perioperative period, even in patients undergoing THA and TKA ^26– 29^. So far, preoperative selection criteria, including preoperative urinary bladder function, have failed to solve the problem, but potentially postoperative catheterization may be avoided or reduced by using a lower-dose spinal anesthesia ^30^. Furthermore, a higher ultrasound-verified bladder volume before catheterization may reduce catheterization, but the literature is inconclusive ^27^. In the two recent fast-track modern anesthesia randomized series ^14, 15^, no differences in need for urinary catheterization with well-defined criteria were found.

In summary, there is a need for large-scale randomized studies with well-defined criteria for urinary bladder catheterization to demonstrate potential differences between the two anesthetic techniques. Importantly, such studies need to provide multimodal opioid-sparing analgesia since opioids may have a negative effect on urinary bladder function ^27^.

## Implications within a fast-track setup

Improvements in overall perioperative care regarding anesthesia, analgesia, fluid management, nursing care, and rehabilitation have led to a pronounced reduction of LOS to about 1 to 3 days with return to home ^11– 15^ and more recently even the potential to perform THA and TKA on an outpatient basis in selected patients ^31– 33^. A common feature of previous randomized studies as well as the large epidemiological studies ^2– 7^ is the lack of detailed information about the perioperative management and the two anesthetic techniques, including patient characteristics. Furthermore, the epidemiological studies rely on diagnostic codes, which may not always be exact. Although a balanced view of all available data from within a reasonably recent time frame may support the use of regional anesthesia, there is a severe need for large-scale prospective randomized controlled trials to compare general versus spinal anesthesia, knowing that the choice of anesthesia represents only one of the many factors that influence outcome. In this context, the focus must include potential identification of subgroups of patients who may or may not benefit from a given anesthetic procedure. Such studies must use an evidence-based approach when choosing the two anesthetic techniques, especially within the context of a fast-track setup with provision of an optimized multimodal, oral opioid-sparing analgesia to facilitate early mobilization and reduce adverse events, including the possibility for early mobilization and urinary bladder dysfunction ^34^. Thus, most previous studies have not included gabapentinoids, which may be appropriate in hip replacement ^35^ but not in knee replacement ^36, 37^, and preoperative high-dose glucocorticoid may provide major analgesic effects with reduced opioid use and side effects ^38, 39^. Furthermore, the use of high-volume LIA is evidence based in TKA but not in THA ^16^. Also, future studies should include early (within a few hours) mobilization, which may be important to reduce thromboembolic complications ^40^ that may be independent of anesthetic technique.

In summary, the recent development of optimized general and regional anesthetic techniques together with advances in multimodal opioid-sparing analgesia combined with the fast-track methodology may provide an opportunity in a large randomized study to answer the old question of whether regional anesthesia is better than general anesthesia ^41^.
